# Bioinductive Collagen Implant Augmentation for the Repair of Chronic Lower Extremity Tendinopathies: A Report of Two Cases

**DOI:** 10.7759/cureus.15567

**Published:** 2021-06-10

**Authors:** Austin M Looney, Luc M Fortier, Joseph D Leider, Brandon J Bryant

**Affiliations:** 1 Department of Orthopaedic Surgery, Georgetown University Medical Center, Washington, DC, USA; 2 Department of Orthopaedic Surgery, Georgetown University School of Medicine, Washington, DC, USA; 3 Department of Orthopaedics, Sports Medicine Division, Inova Fairfax Hospital, Falls Church, USA

**Keywords:** bioinductive implant, patellar tendinopathy, hamstring surgery, bioinduction, chronic tendinopathy, bioinductive augmentation, sports medicine, hamstring tendon, regeneten

## Abstract

In this report, we present two cases of refractory chronic lower extremity tendinopathies treated with collagen bioinductive implant augmentation: a 20-year-old male football player with chronic patellar tendinopathy and a 40-year-old active female with chronic proximal hamstring tendinopathy. We demonstrate that bioaugmentation may represent an effective strategy in the surgical treatment of chronic tendinopathies. Both patients were able to return to their pre-injury activity levels at an accelerated rate.

## Introduction

Chronic tendinopathies can be difficult to treat. Patellar tendinopathy is most commonly reported in athletes who play sports that involve strenuous jumping, particularly elite-level volleyball players [1]. Proximal hamstring tendinopathies occur more frequently in runners and soccer players [2-3]. In both cases, a trial of conservative treatment is warranted, consisting of activity modification, nonsteroidal anti-inflammatory drugs (NSAIDs), and physiotherapy [4-5]. While nonoperative strategies may be successful in a majority of cases, approximately 25-30% of patients are unable to return to their sport with conservative measures alone and may benefit from surgical debridement and repair [6-7]. The use of bioinductive implants has emerged as a promising option in orthopedic surgery to facilitate healing and enhance the strength of tissue repair [8]. These implants act as an artificial structure capable of supporting three-dimensional tissue formation that allows for cell attachment and migration, delivery of biochemical factors, and diffusion of cell nutrients to improve the local tissue environment and promote healing [8-9]. The implants are minimally cross-linked and freeze-dried and are designed to gradually undergo degradation within six months, leaving a layer of new tendon-like tissue; this new tissue decreases peak strain, thereby creating an environment conducive to healing [10].

Regeneten (Smith & Nephew, Inc., Andover, MA) is a bioinductive implant composed of type 1 collagen derived from bovine Achilles tendon, which acts as a highly porous scaffold allowing the attachment of regenerative cells and transport of nutrients and waste. While initial results of Regeneten augmentation of rotator cuff repairs have been promising [11], its utility outside of the shoulder is not well explored. limited to a single case report involving bioaugmentation of surgically managed chronic patellar tendinopathy and technique descriptions without follow-up for hip abductor repairs [12-14]. As with any implant, there is a theoretical risk of an inflammatory or foreign body reaction. However, these risks have not been proven to be an issue in the literature [15-16].

We present two cases where the Regeneten bioinductive implant was utilized for the bioaugmentation of repairs of the refractory chronic patellar tendon and proximal hamstring tendinopathies. Verbal and written consent was obtained from the patients for the publication of this article.

## Case presentation

Patient 1: chronic patellar tendinopathy

**Figure 1:**
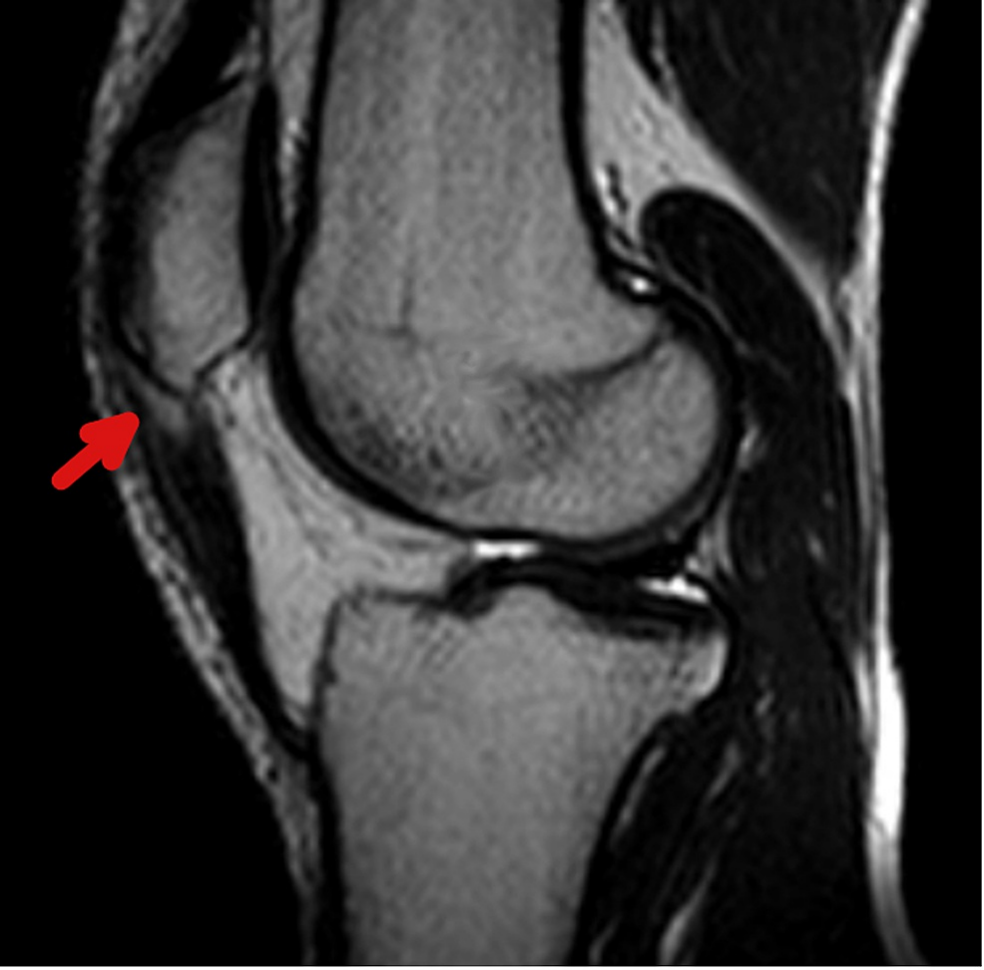
Sagittal T2-weighted MRI of the right knee of the first patient The nidus of tendinopathy is plainly visible just distal to the inferior patellar pole (arrow) MRI: magnetic resonance imaging

A 20-year-old male collegiate football athlete presented with 14 months of atraumatic right knee pain localized to the patellar tendon. His symptoms were exacerbated by activity and had progressed to the point where he was no longer able to play. He denied associated swelling, stiffness, or mechanical symptoms. Examination revealed normal alignment, no effusion, no joint line tenderness, and stable ligaments, with tenderness just distal to the inferior patellar pole. After 11 months of activity modification and conservative treatment failed to alleviate the pain, an MRI of the right knee was performed, which revealed patellar tendinosis with a high-grade partial proximal tear (Figure 1), and the patient elected to undergo debridement and repair with bioinductive patch augmentation.

**Figure 2:**
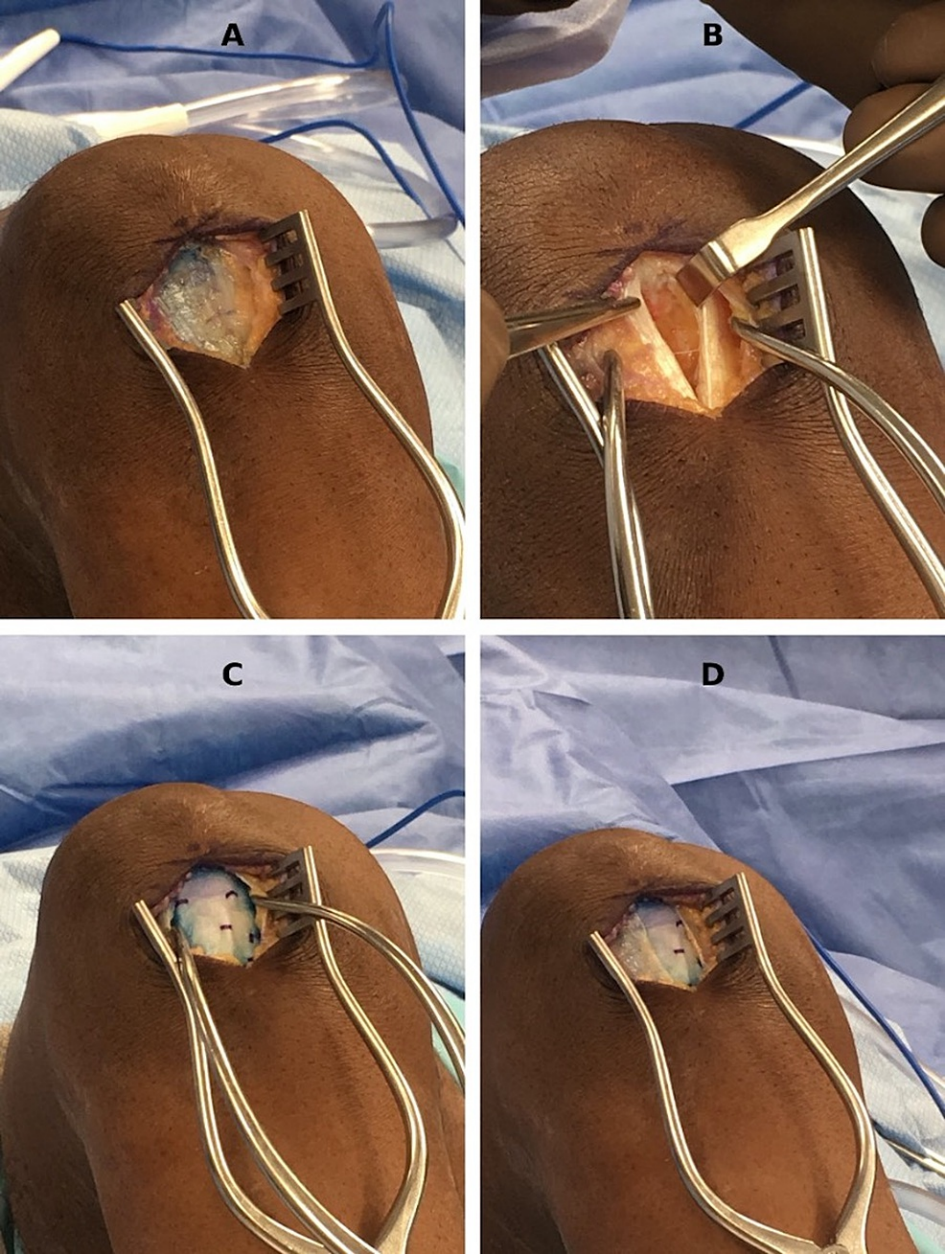
Surgical procedure - patient 1 A small longitudinal midline incision is extended distally from the inferior pole of the patella, and the paratenon layer is identified, sharply divided, and preserved, revealing the area of tendinopathy (A). The tendinopathic tissue is debrided, and the inferior pole of the patella is decorticated to promote the extravasation of marrow elements (B). An appropriately-sized (Smith & Nephew) patch is applied and secured over the area with the included tissue staples (C), and the paratenon is closed over the top (D)

Surgery was performed with a thigh tourniquet through a 2.5-cm longitudinal incision extending distally from the inferior pole of the patella (Figure 2A). The paratenon was divided sharply and preserved, and the tendon was split longitudinally in line with the fibers, exposing the underlying tendinopathic areas, which were sharply excised (Figure 2B). The inferior pole of the patella was decorticated to promote bleeding. A Regeneten bioinductive patch was laid over the tendon and secured with absorbable soft tissue staples (Figure 2C), followed by the closure of the paratenon (Figure 2D).

Postoperatively, the patient was placed in a hinged knee brace in full extension with 45 degrees allowable for passive motion, and he was immediately able to bear weight as far as tolerable. Therapy was initiated within the first week postoperatively, which consisted of quadriceps sets, heel slides, standing toe raises, ankle pumps, and hip abduction. At his first follow-up visit two weeks post-surgery, he reported no pain or any tenderness to palpation. At six weeks postoperatively, he still did not report any pain and was able to begin jogging. By his three-month follow-up, he had completed therapy and had been jogging up to two miles per day with intermittent sprint work. He returned to sport-specific training at six months postoperatively and was cleared to return without restrictions by eight months. By one year postoperatively, he had fully returned to all activities without any pain.

Patient 2: chronic proximal hamstring tendinopathy

**Figure 3:**
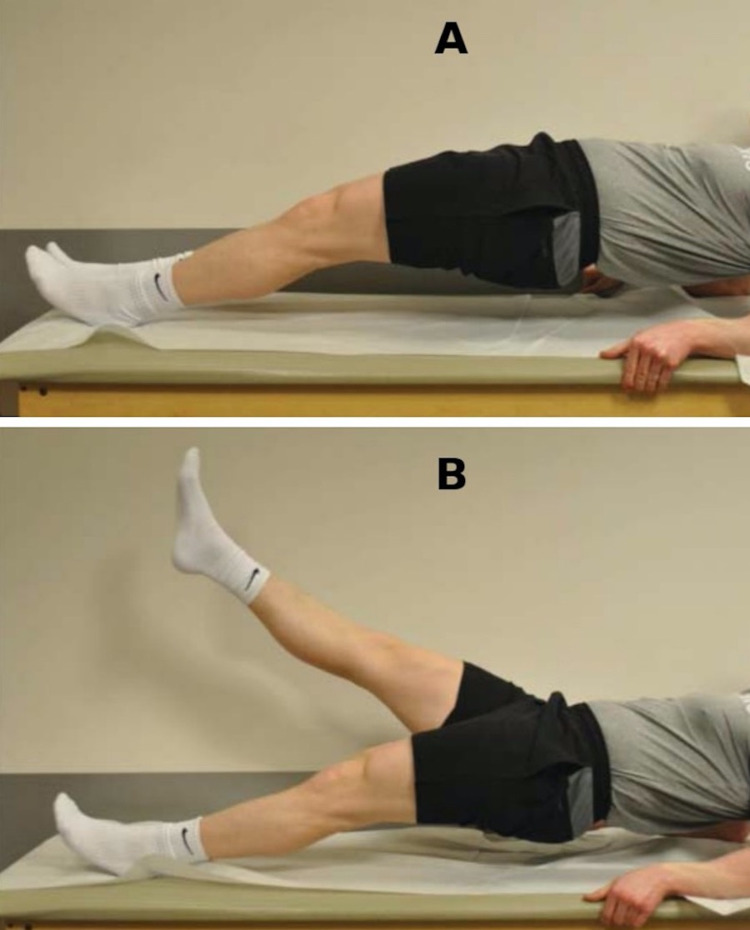
Single-leg bridge or plank test for hamstring pathology The test is performed with the patient supine on the examination table. The patient is instructed to raise the pelvis off the table by extending both hips, supporting himself on his forearms and heels (A). The lower extremity of the unaffected side is then lifted off the table (B) Adapted with permission from Arner et al., J Am Acad Orthop Surg., 2019 [3]

**Figure 4:**
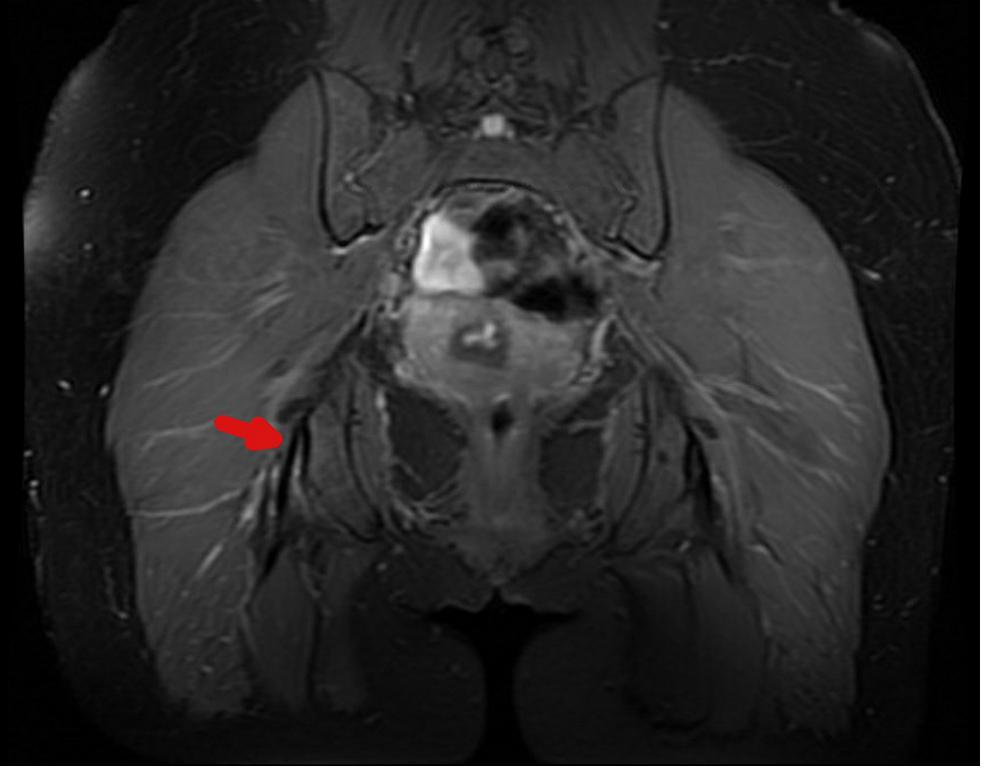
Coronal proton density (PD) fat-saturated (FS) MRI of bilateral hips at the level of the ischial tuberosities and lateral hamstring origins On the right, a high-intensity signal at the origin is consistent with high-grade partial tearing and tendinopathy (arrow) MRI: magnetic resonance imaging

A 40-year-old active female with over 20 years of posterior right hip pain presented for evaluation following an acute exacerbation due to running. Her symptoms were localized to the ischial tuberosity in the region of the hamstring origin. She denied any groin pain, lateral pain, or radicular symptoms. Her pain was exacerbated by most activities and prevented her from exercising. She had previously failed eight months of formal physical therapy, three prolotherapy injections, and three platelet-rich plasma (PRP) injections. Examination revealed normal passive motion without groin pain, impingement signs, or trochanteric tenderness; marked tenderness over the proximal hamstring origin; and reproduction of symptoms with resisted prone knee flexion and plank test (Figure 3, representative image). An MRI of the right hip demonstrated proximal hamstring tendinosis with tearing of the deep fibers of semimembranosus origin (Figure 4). She agreed to undergo operative treatment.

**Figure 5:**
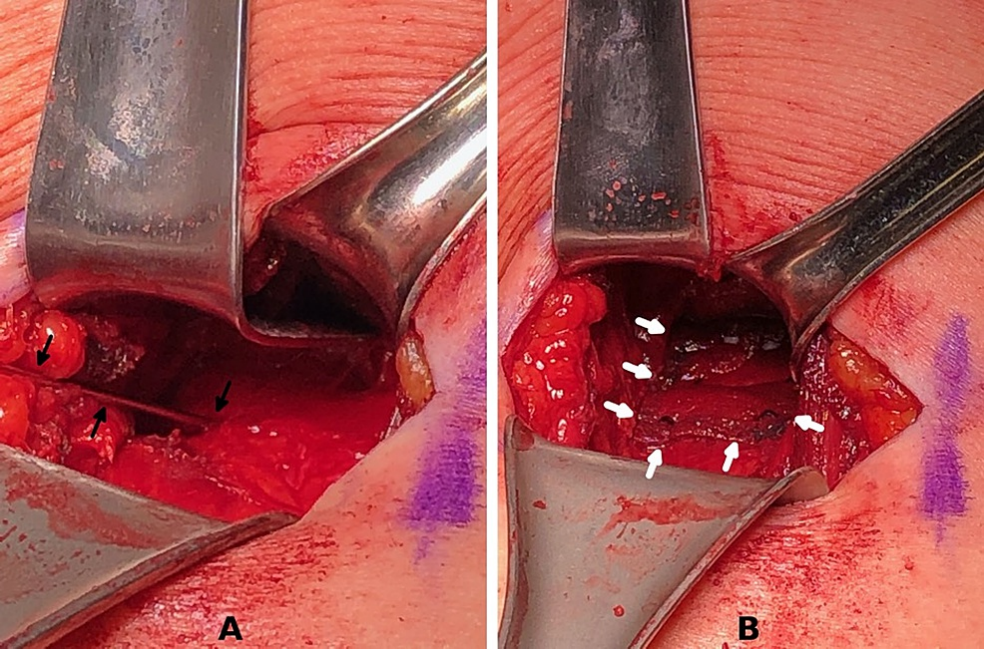
Surgical procedure - patient 2 The hamstring origin is exposed through a small transverse incision in the gluteal fold, retracting the inferior border of the gluteus maximus muscle superiorly. Adhesions and pathologic tissue is identified and debrided. The ischium is drilled with a Kirschner wire (A, black arrows) to promote the extravasation of marrow elements. A Regeneten bioinductive patch is then applied to the site and secured with the included tissue staples (B)

A transverse incision was made within the right gluteal fold. With the gluteus major muscle retracted superiorly, the proximal hamstring fascia was identified and incised at the semimembranosus origin. The pathologic tissue was debrided. Several unicortical holes were drilled into the ischium with a 0.045-inch Kirschner wire to allow extravasation of marrow contents (Figure 5A), and a Regeneten bioinductive patch was placed over the proximal hamstring origin (Figure 5B) and secured to the proximal tendon with the absorbable soft tissue staples. The area was then irrigated and closed.

Postoperatively, the patient was placed into a hinged knee brace locked at 45 degrees for two weeks to protect the repair but was otherwise allowed to bear weight. Therapy was initiated during the first week postoperatively, consisting of ankle pumps and quadriceps sets. The brace was discontinued at the two-week follow-up visit with progressive motion and weight-bearing allowed. At her six-week follow-up, her pain was found to be improving, and she could ambulate without crutches. By nine weeks postoperatively, she was able to perform activities pain-free, which she had not been previously able to do, including the plank test. By 3.5 months postoperatively, she had returned to running and swimming and only experienced pain with prolonged sitting. By five months, she was running up to 3.5 miles three times a week, and her sitting pain had completely resolved. She continued swimming and running pain-free without recurrence with gradual improvement in her pace through her one-year follow-up period.

## Discussion

Traditional surgical options for refractory chronic tendinopathies involve debridement of the involved tendon or peritendinous tissue with osseous drilling or decortication at attachment sites for marrow stimulation, possibly followed by formal repair (Tables 1, 2) [17-21]. However, the recovery period is often prolonged and complicated [22-24], which may be partially due to the fact that subsequent healing must occur under adverse conditions, the same that had contributed to the development of tendinopathy in the first place.

Bioaugmentation may help overcome local tissue deficiencies by promoting an increase of favorable growth factors and cytokines at the repair site [8]. To our knowledge, only one case of patellar tendon repair augmentation with a bioinductive implant has been reported in the literature, by McMillan et al. [12]. The patient was a 37-year-old male, an active-duty Air Force member with a nine-month history of chronic patellar tendonitis that had failed conservative measures. The surgical treatment included debridement of a tendinopathic focus near the patellar origin, decortication of the inferior pole of the patella, and formal suture anchor repair. Unlike in our case, PRP was added to the repair site before placing a Regeneten patch. We elected not to perform a formal repair as our patient’s defect was not full-thickness, and a substantial portion of the tendon remained in continuity with the patella. Likewise, we did not feel that the addition of PRP would offer further benefits beyond the placement of the bioinductive patch.

Postoperatively, McMillan et al. had their patient on crutches for four weeks with partial weight-bearing. Given that we did not perform a formal repair, we allowed our patient to immediately bear weight as far as tolerable. Similar to our rehabilitation protocol, physical therapy was initiated by McMillan et al. within the first week postoperatively with gradual progression to full activity at three months. Sports activities involving jumping were restricted until six months postoperatively, which is also the duration for which we restricted sport-specific training. By way of clinical comparison, the patient in McMillan et al.'s case reported a decrease in Visual Analog Scale for pain from 7 to 2 at six months postoperatively, while our patient reported an absence of pain as early as two weeks postoperatively, at which time he also expressed feeling better than he had felt prior to the surgery. This difference in outcomes may be attributed to the differing extents of the procedures performed, and differences in postoperative protocols.

We are not aware of any reports in the literature about Regeneten augmentation for proximal hamstring repairs to date. These can be difficult injuries to manage [3,5,25]. On average, it takes up to six months for conservatively managed patients to fully recover, while in 20% of patients, symptoms persist and become refractory to nonsurgical modalities [26]. In a cohort of 25 patients with partial-thickness tears who were managed with nonoperative treatment, 40% of the patients eventually required surgical intervention due to refractory pain or limited function [27]. In a systematic review, 99% of patients (n=266) with surgically treated proximal hamstring tendinosis and partial tears were able to return to sports [5], though in some cases recovery was protracted, and some athletes experienced residual symptoms [28-30]. In the series reported by Bowman et al., athletes began “gentle sport-specific activities” at 12 weeks, with an unrestricted return to activity delayed till six months. By comparison, our patient returned to running and swimming by three months postoperatively and was exercising at a higher-than-preinjury level by eight months.

**Table 1:** Previously published reports on surgically treated chronic patellar tendinopathy *Tendons/patients. ^†^Average (range). ^§^To previous level. ^⸸^To sport-specific training (but not necessarily full competition) at previous level. ^‖^Basis for rating scheme not specified. ^¶^Out of 27 professional athletes C: competitive (high school, collegiate, or Olympic); F: female; IKDC: International Knee Documentation Committee; IPP: inferior patellar pole; M: male; P: professional; R: recreational

Lead author	Year	No.*	Patient characteristics					Treatment	Outcomes	Return-to-play	
			Age, years^†^	Sex	Level(s)	Sport(s)				No. (%)^§^	Time^⸸^, months
Ferretti [31]	2002	33/27	26.9 (18-31)	24 M, 3 F	C/P	Basketball, dance, football, skiing, tennis, volleyball		Open debridement, IPP excision and drilling	23 excellent, 5 good, 1 fair, 4 poor^‖^	18 (82)	6
Pascarella [32]	2011	73/64	24.6 (16-35)	40 M, 24 F	R/P	Basketball, running, soccer, tennis, volleyball		Arthroscopic debridement and IPP excision	Lysholm scores^†^: 1 year: 86.4 (-), 3 years: 86.4 (-); IKDC scores^†^: 1 year: 94.7 (-), 3 years: 95.5 (-)	19 (70.4)^¶^	5
Shelbourne [33]	2006	22/16	19.7 (16-25)	10 M, 6 F	C/P	Basketball, football, volleyball, track/field		Open debridement and IPP decortication	11 excellent (return to preinjury level of activity without pain), 3 good (return to preinjury level of activity but with mild to moderate pain), 2 fair (improvement in symptoms but unable to return to preinjury level of sport, or moderate pain)	14 (87.5)	8

**Table 2:** Previously published reports on surgically treated chronic proximal hamstring tendinopathy *Tendons/patients. ^†^Average (range). ^§^To previous level. ^⸸^To sport-specific training (but not necessarily full competition) at previous level. ^‖^Mean ± standard deviation (SD) ADL: activities of daily living; C: competitive (high school, collegiate, or Olympic); F: female; LEFS: Lower Extremity Functional Scale; M: male; NR: not reported; P: professional; R: recreational

Lead author	Year	No.*	Patient characteristics					Treatment	Outcomes	Return-to-play	
			Age, years^†^	Sex	Level(s)	Sport(s)				No. (%)^§^	Time^⸸^, months
Benazzo [34]	2013	17/17	26.6 (20-34)	12 M, 5 F	C/P	Running, soccer, track/field		Open debridement, ischial drilling, sciatic neurolysis	15 excellent (asymptomatic, able to return to same level of sport), 2 good (able to return to same level of sport with pain during intense efforts); Tegner score^†^: 7.8 (7-10)	17 (100)	4
Bowman [29]	2013	17/14	43.3 (19-64)	3 M, 14 F	R/C/P	Bodybuilding, dance, field hockey, running, tennis, track/field, waterskiing		Open debridement, ischial curettage/decortication, formal suture anchor repair	Marx scale^‖^: 6.5 ± 5.3, LEFS^‖^: 73.3 ± 9.9	14 (100)	NR
Lempainen [2]	2009	103/90	34 (16-63)	58 M, 32 F	R/C/P	Aerobics, dance, running, skiing, soccer, tennis, track/field		Open proximal semimembranosus tenotomy at origin, tenodesis to biceps femoris, ± sciatic neurolysis	62 excellent (asymptomatic, able to return to same level of sport), 30 good (able to return to same level of sport with occasional minor symptoms during strenuous sports activity), 5 fair (unable to return to previous level due to persistent symptoms), 6 poor (unable to participate in sports with symptoms affecting ADL)	80 (89)	5

## Conclusions

Bioaugmentation may represent an effective strategy in the surgical treatment of chronic tendinopathies outside of the shoulder, with both of our patients being able to return to their pre-injury activity levels at an accelerated rate. Larger series with long-term follow-ups and subsequent prospective trials are required to better evaluate the clinical implications of this emerging technology.
